# Progress in Skin Diseases

**Published:** 1901-06-15

**Authors:** 


### PROGRESS IN SKIN DISEASES.

(*Continued from page* 150.)

**Blastomycetic Dermatitis.**—Stelwagon[44] describes a case which was at first thought to be tuberculosis. On the backs of the hands was a raised surface with papillæ discharging in some spots a seropurulent and elsewhere a clear gummy fluid. On the arms were several flat masses like very sluggish carbuncles discharging similar fluids. Microscopically a few yeast fungi were found amongst other organisms, but no tubercle bacilli were to be made out at any time. Apparently complete recovery took place under the use of large doses of iodides.

**Roentgen Ray Treatment.**—A case of lupus successfully treated was shown by Pusey at Chicago.[45] The patient's face had been affected for four years, and the disease was in an active state when treatment commenced. Daily exposures were given from May 8th to November 14th, except for rather more than six weeks. All the lesions disappeared by October, and no active trace of the disease remained. A. Everett Smith[46] used a mask of sheet lead which shut off the rays except where a hole, the exact size of the affected area, had been cut. Twelve exposures were made, one every fifth day for 20 minutes, the tube being only two inches from the face. It has been suggested that a special tube to give a light of very short wave length might improve the already good results. Pusey[47] lays down that the current should not be of more than 1½ amperes and 12 volts strength; the inductor should not give more than a 30 c.m. spark. At the commencement of the treatment the sittings should not be longer than five minutes, and the distance of the tube not more than 15 c.m. Evidence that the exposures have been carried far enough is given by the appearance of erythema or pigmentation, and when used for the removal of superfluous hair, by its blanching or loosening. The lesions produced by careless use of the rays may, says the *British Medical Journal*,[48] be a temporary erythema, a dermatitis with vesicles and bullæ and excoriations, or a necrosis with deep ulcerations. There may be shedding of the hair and nails or pigmentation of the skin. The unfortunate effects may only appear some days or even two or three weeks after exposure, and there is also a kind of cumulative effect in their action. Jutassy[49] reports success with X-ray treatment in (*a*) patch of lupus after seven sittings, (*b*) in L. erythematous, (*c*) chronic eczema of the hand, (*d*) hypertrichosis, (*e*) a port wine mark (nævus). Here, however, a white scar was left and some surrounding pigmentation. Stembeck[50] succeeded in getting a rodent ulcer of nine years' duration to heal by prolonged treatment under the rays. R. Bowman[51] describes the treatment of L. erythematosus which gave him very good results. He advises that a trial exposure should be given and a week allowed to elapse to avoid the risk of burns; the tube must not be nearer than 5 inches, and the duration should not exceed 10 minutes. He protects the healthy surfaces by three or four layers of lead foil, gives daily exposures till the skin looks sunburnt, then stops for several days until it is quite pale and has nearly finished scaling.

McMurray,[52] in an old case of lupus, injected increasing doses of tuberculin for three months and applied the X-rays for a similar period afterwards, by which time almost the entire area was healed. He advocates attaching a copper wire to the anodal end of the tube and carrying it near the kathodal end, so as to allow the tube being brought close to the patient without sparking. J. H. Sequeira[53] has treated 12 cases of rodent ulcer with the rays, four of which have healed over, and in all satisfactory progress has been made. The treatment, moreover, is painless. Finsen's light method is being also used with success by Malcolm Morris, who describes the technique in a paper with S. E. Dove.[54] An inflammatory reaction follows an application, and the success seems to depend on this. The difficulties lie in the expense and the small area which can be treated at a time. However, the results are excellent, permanent, and the method painless.

Lupus, rodent ulcer, and L. erythematosus are cured if the diseased area is small, not much scarred or pigmented, and if the diseased parts are not deep below the surface or highly vascular. However, the situation may be one to which the rays cannot be applied, as in the interior of the nose.

**Vaccinal Lupus.**—An interesting case where the disease was situated on a vaccine scar is fully discussed by E. G. Little.[55] The patient was vaccinated from calf lymph which had been glycerinated when less than forty-eight hours old, and no other cases are known to have arisen from other children vaccinated from the same source. The two lower insertions did not heal, but when the scabs fell off a raw nodule was left about six weeks after vaccination. There were a few enlarged axillary glands but no signs of phthisis. When the entire patch of lupus was excised, the patient being then nine years old, the tissue showed typical giant cells, and in a guinea-pig inoculated with a portion, a gland showed numerous bacilli. Whether the lupus was introduced by the vaccine, or the vaccinator, or from outside before the wound was closed is uncertain. Similar tuberculous ulcerations have been noted after wounds, especially after circumcision, the incubation period being from twelve to forty-two days, but it does not seem that tubercle bacilli have ever been found in vaccine lymph, even in that from phthisical persons.

[44] Amer. Jour. Med. Sci., February. [45] Brit. Med. Jour., Jan. 12. [46] Med. Press, Feb. 20. [47] Amer. Jour. Med. Sci., January, p. 121. [48] Brit. Med. Jour., Dec. 1. [49] Brit. Med. Jour., Jan. 5. [50] Brit. Med. Jour., March 2. [51] Austral. Med. Gaz., Jan. 25. [52] Ibid. [53] Brit. Med. Jour. Feb. 9. [54] Ibid., p. 326. [55] Brit. Jour. Dermat., March, p. 81.

